# Benford’s Law and articles of scientific journals: comparison of JCR^®^ and Scopus data

**DOI:** 10.1007/s11192-013-1030-8

**Published:** 2013-05-21

**Authors:** Alexandre Donizeti Alves, Horacio Hideki Yanasse, Nei Yoshihiro Soma

**Affiliations:** 1 National Institute for Space Research, INPE, Av. dos Astronautas, 1.758, Jd. Da Granja, São José dos Campos-SP, 12227–010 Brazil; 2 Federal University of São Paulo, UNIFESP, Rua Talim, 330, Vila Nair, São José dos Campos-SP, 12231–280 Brazil; 3 Aeronautic Institute of Technology, ITA, Praça Marechal Eduardo Gomes, 50, Vila das Acácias, São José dos Campos-SP, 12228–900 Brazil

**Keywords:** Benford’s Law, Articles, JCR^®^, Scopus

## Abstract

Benford’s Law is a logarithmic probability distribution function used to predict the distribution of the first significant digits in numerical data. This paper presents the results of a study of the distribution of the first significant digits of the number of articles published of journals indexed in the JCR^®^ Sciences and Social Sciences Editions from 2007 to 2011. The data of these journals were also analyzed by the country of origin and the journal’s category. Results considering the number of articles published informed by Scopus are also presented. Comparing the results we observe that there is a significant difference in the data informed in the two databases.

## Introduction

Benford’s Law (Benford 1938), also known as the first digit law or law of the leading digits, is a logarithmic probability distribution function for the first significant digits, which can be written as

1 $$ P\left( d \right) = \log_{10} \left( {1 + \frac{1}{d}} \right),\,d = { 1},{ 2}, \, \ldots ,{ 9} $$

where *P* is the probability and *d* is the first significant digit in question. The first significant digit of a number is the first non-zero digit on its extreme left like 7 for 725 and 2 for 0.0239. According to Eq. 1, in a given data set the probability of occurrence of a certain digit as first significant digit decreases logarithmically as the value of the digit increases from 1 to 9. The expected proportions for the first digits are shown in Table 1.

**Table 1:** The expected proportions of Benford’s Law for the first digits

First digit (*d*)	*P*(*d*)
0	–
1	0.3010
2	0.1761
3	0.1249
4	0.0969
5	0.0792
6	0.0669
7	0.0580
8	0.0512
9	0.0458
Total	1

This was first observed in 1881 by American astronomer and mathematician Simon Newcomb (Newcomb 1881), who noted that the first pages in his book of logarithmic tables were more worn than later pages, which indicated that the tables of logarithms were not used in a uniform way. From this he inferred that fellow scientists using the logarithm tables were looking up numbers starting with 1 more often than numbers starting with 2, numbers with first digit 2 more often than 3, and so on.

This law was rediscovered in 1938 by American electrical engineer and physicist Frank Benford and is now known as “Benford’s Law”. Benford analyzed 20 lists of large data sets with a total of 20,229 observations and 10 lists of smaller data sets with a total of 2,968 observations. This lists included the surface areas of rivers, the sizes of populations, physical constants, molecular weights, entries from a mathematical handbook, numbers contained in an issue of a magazine, death rates etc. He found that the digit 1 tends to occurs with probability of ~30 %, much greater than the expected 11.1 % (i.e., one digit out of 9).

Benford’s Law has been extensively applied to a wide variety of natural and man-made data sets, such as numerical data on the country-wise adherent distribution of major world religions (Mir 2012), financial data of religious community (Clippe and Ausloos 2012), fraud detection in scientific publications (Hein et al. 2012), detecting electoral fraud (Beber and Scacco 2012), time series analysis of seismic clusters (Sottili et al. 2012), experimental values of *β*-decay half-lives (Ni et al. 2009) and hydrological data (Nigrini and Miller 2007).

Campanario and Coslado (2011) was the first application of Benford’s Law to scientometric data. They used for this study a sample of number of articles published, citations received to journals and impact factors of journals indexed in the Science Citation Index from 1998 to 2007. They used data published in the JCR^®^ database to Spanish universities available on the Web. They identified the first significant digit of each one of these variables for each year separately, and compared this to the number predicted by Benford’s Law. Citations data followed Benford’s Law in all years studied. However, for the data on the number of articles, there was no compliance with Benford’s Law in any of the years considered. The same occurred with the data for impact factors in almost all years studied.

Recently, Egghe and Guns (2012) used a generalization of Benford’s Law related to the general law of Zipf with exponent *β* > 0 in the data of Campanario and Coslado (2011). They applied nonlinear least squares to determine the optimal *β* and showed that this generalized law fits the data better than the classical Benford’s Law.

The present paper extends the work of Campanario and Coslado (2011). We analyzed the compliance of the number of articles published of journals indexed in the JCR^®^ Science and Social Sciences Editions from 2007 to 2011 with Benford’s Law. We also investigated their compliance with Benford’s Law analyzing the number of articles published according to the journal’s country of origin and to the journal’s category. In addition, we make a comparison with the Scopus data.

## Materials and methods

In this study we used data available in the JCR^®^ database on the web from 2007 to 2011, with separate editions for Science and Social Sciences. All journals indexed in the JCR^®^ with at least 1 article published were included. We also take into consideration the journal’s country origin and the journal’s category.

Initially we identified the first significant digit of the number of articles published in each journal indexed in the JCR^®^, for each year and edition, separately, to calculate the frequency of each digit and we compared it with the number predicted by Benford’s Law.

Then, we carry out the *χ* ^2^ test:

2 $$ \chi^{2} \left( {n - 1} \right) = \mathop \sum \limits_{i = 1}^{n} \frac{{\left( {N_{\text{o}} \left( d \right) - N_{\text{e}} \left( d \right)} \right)^{2} }}{{N_{\text{e}} \left( d \right)}},$$

to test the *Null Hypothesis*, *H*_0_ that the observed distribution of the first significant digit, in each case we consider, is the same as the expected number based on Benford’s Law.

For *n* = 9 we have n − 1 = 8 degrees of freedom, and *χ* ^*2*^(8) = 15.507 for a 95 % confidence level. This is the critical value for the acceptance or rejection of the *Null Hypothesis*, that is, if the calculated value of *χ* ^*2*^ is less than the critical value then we accept *H*_0_ and conclude that data is in compliance with Benford’s Law, otherwise, we reject *H*_0_.

Alternatively we can test each of the nine proportions separately. The *Z*-statistic is the test to verify whether the observed proportion for a digit differs significantly from the expected value based on Benford’s Law (Nigrini 2012). The *Z*-statistic formula takes into account the absolute magnitude (the numeric distance) of the difference between the observed and the expected values, the cardinality of the data set, and the expected proportion value and is given by the following equation:

3 $$ Z = \frac{{\left| {P_{\text{o}} - P_{\text{e}} } \right| - \left( \frac{1}{2N} \right)}}{{\sqrt {\frac{{P_{\text{e}} \left( {1 - P_{\text{e}} } \right)}}{N}} }}, $$

where *P*_o_ denotes the observed proportion value, *P*_e_ the expected proportion value, and *N* is the total numbers of observations. The term in the numerator (1/2*N*) is a continuity correction term and it is considered only when it is smaller than the other term in the numerator. For a significant level of 5 %, the cutoff level is 1.96. When *Z*-statistic exceeds 1.96 it indicates that the difference between the observed proportion and the expected proportion values is significant at the 0.05 level, which means there is only a 5 % probability that the difference is due to chance alone.

Data available in the Scopus database were also used. Using this database, we tested the number of articles published in journals of some countries and categories of the JCR^®^. Similarly, all journals indexed in Scopus with at least 1 article published were considered. Furthermore, only journals present in both databases were considered.

Using the binomial distribution (Ni et al. 2009), the expected root-mean-square error, ∆[*N*(*d*)]:

4 $$ \Updelta N\left( d \right) = \sqrt {NP\left( d \right)\left( {1 - P\left( d \right)} \right),} $$

was also calculated where *N* is the total number of points considered and *P*(*d*) is the prediction of Benford’s Law.

## Results and discussions

Campanario and Coslado (2011) noted that the number of articles published, citations received to journals and impact factors of journals indexed in the JCR^®^ Science Edition from 1998 to 2007 not always are in compliance with Benford’s Law. A summary of their analysis is presented in Table 2.

**Table 2:** *χ* ^*2*^ values for the number of articles published, citations received and impact factors of journals indexed in the JCR^®^ Science Edition from 1998 to 2007 (from Campanario and Coslado (2011))

Year	Articles	Citations	Impact factors
1998	27.8*	15.1	6.6
1999	27.4*	7.1	11.3
2000	16.2*	4.5	22.2*
2001	38.1*	5.2	20.2*
2002	57.9*	3.1	24.9*
2003	43.5*	3.5	12.5
2004	31.3*	3.0	16.7*
2005	41.5*	11.2	16.3*
2006	27.8*	9.7	39.3*
2007	31.3*	8.4	40.4*

* Denotes significant difference between observed and expected values at *p* = 0.05

Observe that *χ* ^*2*^ values for the number of articles are greater that the critical value (15.507) in all years, that is, all of them are not in compliance with Benford’s Law.

We decided to extend their analysis and we investigated the data of the following years. We analyzed the number of articles published in journals indexed in the JCR^®^ Science Edition from 2007 to 2011 and the results are shown in Table 3. Despite of the fact that they had already calculated the *χ* ^*2*^ value for 2007, we calculated it again for the sake of verifying the compatibility of our results with theirs. We observed a small difference probably due to the fact that we considered a larger number of journals, with the update of the JCR^®^ database.

**Table 3:** The frequency of occurrence of the figure *d* as the first significant digit, obtained from the number of articles published of journals indexed in the JCR^®^ Science Edition from 2007 to 2011

Year	*d*	1	2	3	4	5	6	7	8	9	Total	*χ* ^*2*^
2007	*N*_o_	1,730	1,047	840	643	515	450	389	354	307	6,275	31.6*
	*N*_e_	1,888.8	1,105.0	783.7	608.0	497.0	419.8	364.0	321.3	287.4		
	*∆N*(*d*)	36.34	30.17	26.19	23.43	21.39	19.79	18.52	17.46	16.56		
	*Z* value	4.36**	1.90	2.12**	1.47	0.82	1.49	1.33	1.86	1.17		
2008	*N*_o_	1,790	1,090	841	670	595	415	411	343	332	6,487	41.1*
	*N*_e_	1,952.6	1,142.4	810.2	628.6	513.8	434.0	376.2	332.1	297.1		
	*∆N*(*d*)	36.94	30.68	26.63	23.83	21.75	20.12	18.82	17.75	16.84		
	*Z* *value*	4.39**	1.69	1.13	1.71	3.72**	0.93	1.82	0.60	2.06**		
2009	*N*_o_	2,018	1,204	955	766	631	494	430	402	316	7,216	34.3*
	*N*_e_	2,172.0	1,270.7	901.3	699.2	571.5	482.8	418.5	369.5	330.5		
	*∆N*(*d*)	38.96	32.36	28.08	25.13	22.94	21.23	19.86	18.72	17.76		
	*Z* value	3.95**	2.05**	1.88	2.63**	2.58**	0.49	0.56	1.73	0.77		
2010	*N*_o_	2,172	1,321	1,065	798	720	554	472	406	367	7,875	44.9*
	*N*_e_	2,370.4	1,386.8	983.6	763.1	623.7	526.8	456.7	403.2	360.7		
	*∆N*(*d*)	40.71	33.80	29.34	26.25	23.96	22.17	20.74	19.56	18.55		
	*Z* value	4.87**	1.93	2.75**	1.31	4.00**	1.19	0.71	0.14	0.33		
2011	*N*_o_	2,246	1,371	1,070	880	694	591	495	428	353	8,128	43.2*
	*N*_*e*_	2,446.5	1,431.3	1,015.2	787.6	643.7	543.8	471.4	416.2	372.3		
	*∆N*(*d*)	41.35	34.34	29.81	26.67	24.35	22.53	21.07	19.87	18.85		
	*Z* *value*	4.84**	1.74	1.81	3.44**	2.05**	2.06**	1.10	0.59	0.98		

* Denotes significant difference between observed and expected values at *p* = 0.05

** Denotes significant difference between the observed proportion and the expected proportion at the 0.05 level

The *χ* ^*2*^ values in all years are significantly greater than the critical value. Furthermore, we observe that the *Z* values for digit 1 are greater than the cutoff level (1.96) in all years. The same occurred with digit 5, except in 2007.

Campanario and Coslado (2011) take into consideration only journals of the Science Edition but we extended the calculation for the JCR^®^ Social Sciences Edition. The result is presented in Table 4 and, as can be seen, the result is even worse. All years are not in compliance with Benford’s Law and the *Z* values are greater than the cutoff level for almost all digits. They mentioned in their paper that they have no explanation for these differences.

**Table 4:** The frequency of occurrence of the figure *d* as the first significant digit, obtained from the number of articles published of journals indexed in the JCR^®^ Social Sciences Edition from 2007 to 2011

Year	*d*	1	2	3	4	5	6	7	8	9	Total	*χ* ^*2*^
2007	*N*_o_	406	492	336	227	125	98	55	51	43	1,833	263.1*
	*N*_e_	551.7	322.8	228.9	177.6	145.2	122.6	106.3	93.9	84.0		
	*∆N*(*d*)	19.64	16.31	14.15	12.66	11.56	10.70	10.01	9.43	8.95		
	*Z* value	7.40**	10.35**	7.52**	3.86**	1.70	2.26**	5.08**	4.48**	4.51**		
2008	*N*_o_	438	492	392	242	134	91	58	48	57	1,952	287.2*
	*N*_e_	587.6	343.7	243.8	189.2	154.6	130.6	113.2	99.9	89.4		
	*∆N*(*d*)	20.27	16.83	14.61	13.07	11.93	11.04	10.33	9.74	9.24		
	*Z* value	7.36**	8.78**	10.10**	4.00**	1.68	3.55**	5.30**	5.28**	3.45**		
2009	*N*_o_	507	563	425	262	158	118	75	54	53	2,215	289.3*
	*N*_e_	666.7	390.1	276.7	214.6	175.4	148.2	128.5	113.4	101.4		
	*∆N*(*d*)	21.59	17.93	15.56	13.92	12.71	11.76	11.00	10.37	9.84		
	*Z* value	7.38**	9.62**	9.50**	3.36**	1.33	2.53**	4.81**	5.67**	4.87**		
2010	*N*_o_	622	722	487	302	180	141	88	72	54	2,668	364.5*
	*N*_e_	803.1	469.8	333.2	258.5	211.3	178.5	154.8	136.6	122.2		
	*∆N*(*d*)	23.69	19.67	17.08	15.28	13.95	12.91	12.07	11.38	10.80		
	*Z* value	7.62**	12.79**	8.97**	2.81**	2.21**	2.87**	5.49**	5.62**	6.26**		
2011	*N*_o_	674	794	523	328	205	137	91	76	63	2,891	409.8*
	*N*_e_	870.2	509.1	361.1	280.1	229.0	193.4	167.7	148.0	132.4		
	*∆N*(*d*)	24.66	20.48	17.78	15.90	14.52	13.43	12.57	11.85	11.24		
	*Z* value	7.94**	13.89**	9.07**	2.98**	1.61	4.17**	6.06**	6.03**	6.12**		

* Denotes significant difference between observed and expected values at *p* = 0.05

** Denotes significant difference between the observed proportion and the expected proportion at the 0.05 level

Mir (2012) observed that the data of three major Christian denominations follow Benford’s Law. However, when Christianity is considered as a single religious group, the distribution of the significant digits of the adherent data deviates from the predictions of Benford’s Law. Inspired by this observation we analyzed the journals according to their country of origin and to their JCR^®^ category.

Table 5 presents the total number of countries that are in compliance (YES) or not (NO) with Benford’s Law considering the *χ* ^*2*^ values for the number of articles published in journals indexed in the JCR^®^ Science Edition from 2007 to 2011, highlighting the three countries with the highest *χ* ^*2*^ values that are not in compliance with Benford’s Law and their respective number of journals and articles considered in each year.

**Table 5:** Total number of countries that are in compliance (YES) or not (NO) with Benford’s Law considering the *χ* ^*2*^ values for the number of articles published in journals indexed in the JCR^®^ Science Edition from 2007 to 2011

Year	YES (%)	NO (%)	Country	No. of journals (no. of articles)	*χ* ^*2*^
2007	56 (81.16)	13 (18.84)	Turkey	7 (464)	41.9*
			Slovakia	10 (540)	27.1*
			Croatia	10 (578)	22.7*
2008	61 (84.72)	11 (15.28)	Ukraine	4 (309)	44.6*
			United States	2461 (405,322)	26.9*
			Uruguay	1 (8)	18.8*
2009	71 (92.21)	6 (7.79)	Poland	101 (7,642)	26.2*
			United States	2,551 (413,409)	23.7*
			Finland	13 (927)	22.9*
2,010	68 (81.93)	15 (18.07)	Poland	120 (8,936)	31.2*
			Turkey	47 (3,396)	29.8*
			Singapore	50 (4,790)	23.7*
2011	67 (82.72)	14 (17.28)	Poland	124 (9,721)	40.5*
			Turkey	52 (3,953)	29.5*
			Switzerland	170 (26,609)	25.3*

* Denotes significant difference between observed and expected values at *p* = 0.05

It is possible to observe that the great majority of the countries is in compliance with Benford’s Law. “Poland” and “Turkey” are the countries that appeared more times in the list of countries that are not in compliance with Benford’s Law. In the case of “Turkey” it is interesting to note that the number of journals indexed in the JCR^®^ greatly increased from one year to another. Furthermore, one can see that the *χ* ^*2*^ values decrease as the number of journals and articles increases. It is worth observing that the number of journals indexed in the JCR^®^ is very small for some countries, not being sufficient for using the *χ* ^2^ test for the adherence of the data to Benford’s Law. According to Nigrini (2012), the rule for Benford’s Law for first non-zero significant digit *χ* ^2^ test is that the expected number of observations of each cell should be at least 5, hence, the number of observations should be at least 100 (100 times 0.0458 which is close enough to 5).

The result is very similar for the journals indexed in the JCR^®^ Social Sciences Edition. Only a few countries are not in compliance with Benford’s Law, as shown in Table 6. Nevertheless, the *χ* ^*2*^ values are much smaller than the values presented when journals were considered as a single group. It is interesting to observe that “United States” and “England” are not in compliance with Benford’s Law in all years.

**Table 6:** Total number of countries that are in compliance (YES) or not (NO) with Benford’s Law considering the *χ* ^*2*^ values for the number of articles published in journals indexed in the JCR^®^ Social Sciences Edition from 2007 to 2011

Year	YES (%)	NO (%)	Country	No. of journals (no. of articles)	*χ* ^*2*^
2007	38 (92.68)	3 (7.32)	United States	999 (44,124)	134.5*
			England	464 (23,285)	123.2*
			Netherlands	116 (6,979)	18.2*
2008	42 (95.45)	2 (4.55)	United States	1,042 (46,559)	157.2*
			England	484 (25,237)	135.8*
2009	45 (88.24)	6 (11.76)	United States	1,067 (48,548)	199.2*
			England	545 (27,829)	89.1*
			Turkey	7 (274)	25.1*
2010	47 (90.38)	5 (9.62)	United States	1,199 (53,586)	187.8*
			England	716 (35,160)	124.5*
			Finland	2 (87)	19.1*
2011	44 (83.02)	9 (16.98)	United States	1,254 (57,695)	185.0*
			England	828 (40,470)	153.5*
			Finland	2 (114)	23.0*

* Denotes significant difference between observed and expected values at *p* = 0.05

Other analysis carried out took into consideration the journal’s category in the JCR^®^ Science Edition from 2007 to 2011. The result is presented in Table 7. It is possible to verify that the percentage of categories that are in compliance with Benford’s Law is larger compared to the percentage of countries that are in compliance with Benford’s Law in almost every year, except in 2009. “Mathematics” and “Nursing” appeared more times in the list of categories that are not in compliance with Benford’s Law.

**Table 7:** Total number of journal’s categories that are in compliance (YES) or not (NO) with Benford’s Law considering the *χ* ^*2*^ values for the number of articles published in journals indexed in the JCR^®^ Science Edition from 2007 to 2011

Year	YES (%)	NO (%)	Category	No. of journals (no. of articles)	*χ* ^*2*^
2007	156 (90.70)	16 (9.30)	Statistics & Probability	90 (6,512)	31.0*
			Mathematics	199 (16,141)	25.1*
			History & Philosophy of Science	35 (1,007)	22.4*
2008	151 (87.28)	22 (12.72)	Mathematics, Interdisciplinary Applications	74 (6,103)	27.5*
			Nursing	61 (3,706)	25.9*
			Mathematics	208 (17,228)	24.9*
2009	157 (90.75)	16 (9.25)	Nursing	72 (4,232)	32.1*
			Entomology	72 (4,988)	26.1*
			Statistics & Probability	100 (6,844)	25.4*
2010	159 (91.38)	15 (8.62)	Nursing	88 (5,246)	31.1*
			Mathematics	269 (20,049)	29.8*
			Mathematics, Applied	232 (20,998)	27.7*
2011	157 (89.20)	19 (10.80)	Mathematics, Applied	240 (21,860)	36.0*
			Mathematics	281 (20,961)	31.9*
			Nursing	98 (5,601)	29.1*

* Denotes significant difference between observed and expected values at *p* = 0.05

For the journals indexed in the JCR^®^ Social Sciences Edition, the result is significantly worse compared to the results with journal’s country of origin, as shown in Table 8. In some cases the numbers of journals in compliance with Benford’s Law were lower than the number of journals not in compliance. “Sociology” is a category that is not in compliance with Benford’s Law in all years.

**Table 8:** Total number of journal’s categories that are in compliance (YES) or not (NO) with Benford’s Law considering the *χ* ^*2*^ values for the number of articles published in journals indexed in the JCR^®^ Social Sciences Edition from 2007 to 2011

Year	YES (%)	NO (%)	Category	No. of journals (no. of articles)	*χ* ^*2*^
2007	38 (69.09)	17 (30.91)	Sociology	94 (3,099)	46.9*
			Economics	191 (9,245)	44.2*
			Political Science	89 (3,672)	36.1*
2008	30 (53.57)	26 (46.43)	Economics	206 (10,724)	48.3*
			Law	101 (3,049)	41.3*
			Sociology	98 (3,342)	39.7*
2009	32 (58.18)	23 (41.82)	Sociology	111 (3,581)	49.5*
			Law	113 (3,309)	46.6*
			Economics	246 (11,856)	40.0*
2010	26 (46.43)	30 (53.57)	Sociology	128 (4,159)	59.1*
			Education & Educational Research	180 (6,862)	50.8*
			Political Science	140 (5,078)	46.5*
2011	26 (46.43)	30 (53.57)	Sociology	132 (4,553)	66.6*
			Economics	314 (15,327)	51.7*
			Political Science	145 (5,097)	46.0*

* Denotes significant difference between observed and expected values at *p* = 0.05

It is interesting to observe that the *χ* ^*2*^ values observed for the journals indexed in the JCR^®^ Social Sciences Edition are always greater than those presented for journals indexed in the JCR^®^ Science Edition.

We compare next the number of articles published informed in the JCR^®^ and the Scopus databases. We limited the comparison to journals of some countries and of some categories. To make the comparison, we considered only journals that were present in both databases.

The analysis performed showed that there are some cases where Scopus data are in compliance with Benford’s Law but the JCR^®^ Editions data are not. Also the opposite was observed, that is, where JCR^®^ Editions data are in compliance with Benford’s Law but the Scopus data are not. In Table 9 we summarize these findings with 8 examples.

**Table 9:** Comparison of the number of articles published in indexed journals in the JCR^®^ and the Scopus databases and their compliance with Benford’s Law

2008	JCR^®^ Science Edition		Country: Switzerland		Journals: 145 (152)	
		Articles	Min	Max	*χ* ^*2*^	*Z* value (*d*)
	JCR^®^	22,735	1	1,960	20.1*	3.70** (5)
	Scopus	21,353	1	1,885	11.3	
2007	JCR^®^ Social Sciences Edition		Country: Netherlands		Journals: 116 (116)	
		Articles	Min	Max	*χ* ^*2*^	*Z* value (*d*)
	JCR^®^	6,979	7	325	18.2*	2.70** (2)
	Scopus	6,784	3	318	8.6	
2008	JCR^®^ Science Edition		Country: Japan		Journals: 165 (175)	
		Articles	Min	Max	*χ* ^*2*^	*Z* value (*d*)
	JCR^®^	21,409	9	1,963	6.8	
	Scopus	20,045	5	1,948	17.6*	3.03** (4); 2.22** (9)
2011	JCR^®^ Social Sciences Edition		Country: Germany		Journals: 109 (118)	
		Articles	Min	Max	*χ* ^*2*^	*Z* value (*d*)
	JCR^®^	3,226	2	141	12.0	2.09^**^ (2)
	Scopus	3,020	2	127	18.7*	2.02^**^ (1); 2.09^**^ (2); 1.98^**^ (7)
2011	JCR^®^ Science Edition		Category: Endocrinology & Metabolism		Journals: 115 (122)	
		Articles	Min	Max	*χ* ^*2*^	*Z* value (*d*)
	JCR^®^	15,281	5	704	16.2^*^	2.01** (3); 2.54** (6)
	Scopus	13,164	2	639	7.5	
2011	JCR^®^ Social Sciences Edition		Category: Business		Journals: 110 (113)	
		Articles	Min	Max	*χ* ^*2*^	*Z* value (*d*)
	JCR^®^	4,819	9	273	21.6*	2.62** (1); 3.04** (2)
	Scopus	4,757	6	320	14.0	
2011	JCR^®^ Science Edition		Category: Computer Science, Information Systems		Journals: 132 (135)	
		Articles	Min	Max	*χ* ^*2*^	*Z* value (*d*)
	JCR^®^	9,232	4	564	15.4	2.47** (8)
	Scopus	8,389	4	520	16.3*	2.66** (7)
2010	JCR^®^ Social Sciences Edition		Category: Public, Environmental & Occupational Health		Journals: 112 (116)	
		Articles	Min	Max	*χ* ^*2*^	*Z* value (*d*)
	JCR^®^	9,215	15	485	9.7	
	Scopus	8,635	5	511	15.7*	2.43** (3)

* Denotes significant difference between observed and expected values at *p* = 0.05

** Denotes significant difference between the observed proportion and the expected proportion at the 0.05 level

The examples presented in Table 9 were carefully chosen so that the total number of journals is more than 100. In each example, the number of journals indexed in both databases is presented. Beside this value, we present the number of journals indexed in JCR^®^ database in parenthesis. The columns “Min” and “Max” indicate the minimum and maximum number of articles published in journals indexed in the JCR^®^ and the Scopus databases, respectively, according to the country of origin or category considered. The *χ* ^*2*^ values are also presented, and the values that are not in compliance with Benford’s Law are highlighted. The digits (*d*) with significant differences according to the *Z*-statistic test are also presented. We observed that there are two examples in compliance with Benford’s Law according to the *χ* ^2^ test but with one digit with significant difference according to its *Z* value.

## Conclusions

In this paper we applied Benford’s Law to the data of JCR^®^ Science and Social Sciences Editions, and Scopus, taking into consideration the number of articles published by journals indexed in the two databases. The data of these journals were analyzed by the country of origin and the journal’s category. From the country of origin analyses the majority is in compliance with Benford’s Law. In the case of journal’s category, the majority also follows Benford’s Law, except two recent years (2010 and 2011) in journals indexed in the JCR^®^ Social Sciences Edition.

The nonconformity with Benford’s Law identified with the analysis performed in this work could be indications of either incomplete data (for instance, Karamourzov (2012) observed that there is a small fraction (<8 %) of journals of Russia indexed by JCR^®^ in 2010; Michels and Schmoch (2012) noted the steady increase in recent years of publications that have been indexed at Web of Science and Scopus too), data errors, inconsistencies, or anomalies, and/or conformity to a large exponential power law, occurring with the JCR^®^ and/or SCOPUS data, in view of significant differences observed. These indications were already mentioned in previous works where nonconformities were observed (see, for instance, Nigrini (2012)).

We believe that the main contribution of this study is to alert about these differences and, perhaps, provide an explorative instrument to identify where possibly some data anomalies may be occurring, regardless of which database is correct.
